# Effectiveness of Short-Term Health Coaching on Diabetes Control and Self-Management Efficacy: A Quasi-Experimental Trial

**DOI:** 10.3389/fpubh.2019.00314

**Published:** 2019-10-30

**Authors:** Ruey-Yu Chen, Li-Chi Huang, Chien-Tien Su, Yao-Tsung Chang, Chia-Lin Chu, Chiao-Ling Chang, Ching-Ling Lin

**Affiliations:** ^1^School of Public Health, Taipei Medical University, Taipei, Taiwan; ^2^Endocrinology and Metabolism, Cathay General Hospital, Taipei, Taiwan; ^3^Department of Family Medicine, Taipei Medical University Hospital, Taipei, Taiwan

**Keywords:** diabetes, health coaching, self-efficacy of diabetes self-management, HbA1c, diabetes shared care network

## Abstract

**Introduction:** The aim of this study was to explore the effectiveness in HbA1c lowering and self-efficacy of diabetes self-management of a 6 months coaching intervention.

**Methods:** This paper was a two-armed coaching intervention study in which 116 participants who presented type 2 diabetes were recruited at a medical center. The intervention group had health coaching and usual care for 6 months, whereas the control had usual care only. The main outcome variables were HbA1c level and self-efficacy of diabetes self-management, in followed-up measure at 3 and 6 months.

**Results:** We found that an approximate 0.68% (CI = 0.40 to 0.96) reduction in HbA1c was achieved after a 6-month health coaching. Both physical activity and self-efficacy of diabetes self-management were shown to benefit by health coaching.

**Conclusions:** Health coaching might be an effective strategy to enhance self-management for diabetes patients in Taiwan where “Diabetes Shared Care Network” had been implemented for over 20 years. Consider limitations of this study, more studies with designs that yield higher quality evidence for the role of health coaching in diabetic patients are needed.

**Clinical Trial Registration:** www.isrctn.com (ID number: ISRCTN52454940, date: 10 May, 2018, retrospectively registered).

## Introduction

Diabetes is a serious chronic disease that affects people worldwide. According to a report by the World Health Organization (WHO), the global prevalence of diabetes doubled between 1980 and 2014 and is ~8.5% in the adult population in 2017 (1). Globally, diabetes caused an estimated 1.5 million deaths in 2012; in addition to mortality, it also causes innumerable economic losses to the medical system and substantial burdens for people with the disease and their families. WHO noted that preventing and managing diabetes should be a priority in every country.

Diabetes is the fifth leading cause of death in Taiwan; with a prevalence of 11.8% in the adult population, but this figure has been rising and is expected to continue to increase (2). What's more, the cost of dialysis is the highest in Taiwan's national medical expenditure, with more than half resulting from the uncontrolled diabetes. In 1997, Taiwan established the “Diabetes Shared Care Network,” which integrated nationwide medical institutions and adjusted health insurance payments for diabetes care (3). After the patients are recruited and joined the diabetes shared care network, they can use a certain number of free services among participating institutions e.g., health education, blood sugar, blood pressure, and blood lipids test, and foot and eye check every year. As the size of the aging population is growing, the prevalence of diabetes is on the rise, so the disease is likely to continue to cause medical and economic burdens. In preventing and treating of diabetes, promoting a healthy lifestyle is an eminent part. However, lifestyle change counseling is seldom applied in diabetes prevention and treatment system. Hence, this kind of studies might be crucial for future application possibilities.

In recent decades, health and wellness coaching has become a new technique used in the care and management of chronic diseases around the world. Health coaching is a patient-centered, patient-decided approach to disease management (4). Although there are no gold standards for the definition of health coaches, standardized training methods and intensity, and intervention methods, some system-review and meta-analysis literature found health coaching intervention had positive effect on type 2 diabetes management (5–7). In many studies, after participation in health coach intervention, most diabetes patients achieved positive results, such as lowered levels of hemoglobin A1c (HbA1c) and decreased body mass index (BMI) (8–20). Health coaching also improves patients' self-efficacy of diabetes self-management and healthy lifestyle (7, 21–26). Therefore, it is in our interest to carry out tests where no health coach clinical studies have been conducted and to explore its practicability and effectiveness in Taiwan.

In this paper, we designed a quasi-experimental test to evaluate the effect of a health coach intervention on diabetes patients' blood sugar management and self-efficacy by a certified coach. The aim of this study was to enhance patients' self-efficacy in diabetes care, including medical compliance and health behavior, and to improve the indicators of diabetes, especially the value of HbA1c.

## Methods

This study was a 6-month-long coaching intervention study, a two-armed, quasi-experimental trial that was approved by the Institutional Review Board (IRB) of Cathay General Hospital. As the intervener and the data analyst was the same person in this study, only physicians responsible for referring potential participants were blinding. After providing signed informed consent, the researcher matched pair each participant between the two setting in terms of gender and age: (1) coaching intervention every 2 weeks on top of diabetes shared care or (2) diabetes shared care only. Data were collected at the baseline and at the end of 3- and 6-months intervention to evaluate and analyze the outcome difference comparing to the baseline. We constructed this article in accordance with the standards of the TREND guidelines.

### Study Procedure

#### Study Population and Recruitment

Participants were recruited from Cathay General Hospital in Taipei, Taiwan. Cathay General Hospital is an 825-bed “Medical Center,” the highest level of hospital accreditation in Taiwan. Two physicians who specialize in endocrine and metabolic disorders screened potential patients with type 2 diabetes mellitus. If a patient met the inclusion criteria, the doctors would then explain the study and invite the participants to join the program. To be considered for inclusion, participants had to be between 20 to 75 years old, diagnosed with type 2 diabetes for at least 1 year, and had an HbA1c of 7.0% or greater for the past 6 months. Individuals with type 1 diabetes mellitus were excluded since they were not the main intervention target for this study. In addition, individuals who were pregnant or trying to get pregnant during the study, had participated in another similar program in the last 6 months, had clinically significant depression or cognitive dysfunction, or failed to sign informed consent were excluded. Participant enrolment was carried out from June 2017 to October 2017. Data collection was conducted from June 2017 to July 2018.

#### Sample Size

To determine a clinically meaningful difference in coaching intervention, defined as a 1% between-group difference in HbA1c and standard deviation of 1.7, with a probability of a type I error of 0.05 and a power of 80%, a total of 47 participants were required to complete the study in each group. Assuming a 20% dropout rate, the target recruitment number was 60 patients in each study group.

### Intervention

#### Intervention Arm: Usual Care and Health Coaching

Coaching intervention was based on Wolever's design but with a longer interval between coaching sessions (27) of 2 weeks. The coaching was provided by a single coach: the coach had over 120 h certified coach training, received the International Coach Federation's (ICF) Associate Certified Coach (ACC) credential and master's-level degrees in public health. The ICF's ACC credential requires that applicants must receive at least 60 h certified coach training, pass a skill check, and actually perform at least 100 h of coaching. Participants in the coaching group had an initial face-to-face session after they signed informed consent for the study and finished baseline testing. The participants were then offered 10- to 20-min telephone coaching sessions biweekly for six sessions within first 3 months and three sessions within last 3 months. Based on ICF's coach definition and related review literature (4, 6, 28), our coach applied the following theoretical techniques: motivational interviewing, transtheoretical model, appreciative inquiry, self-determination theory and design thinking, and also met ICF's coaching competency.

In the first session, the coach asked each participant to establish his or her 6-month HbA1c goal and overall health plan. Then, the coach asked each patient to create one specific behavior goal related to physical activity, healthy diet, medical adherence, and/or regular self-blood glucose monitoring to be addressed during the first 3 months of the study. If a patient had more than one behavior change target, the coach would ask him or her to prioritize the goals and then, together, they addressed one subject per coaching session. The patients needed to design “SMART” (i.e., specific, measurable, attainable, realistic, and timely) goals for their action plans. During the first coaching session, the coach ascertained what was important to each patient about his or her diabetes management, assessed the patient's knowledge about diabetes complications, compiled the history of diabetes management, and identified challenging areas in which the patient would likely require support. Also, a decision-coaching protocol was developed to reinforce the values and responsibilities of self-management, as well as the purpose of coaching intervention.

Most coaching call was made while the participant was at home or during a working lunch break. At the end of each coaching session, the coachee needed to discuss the time, and agenda of the next coaching call with the coach. During each telephone coaching session, the participant reviewed his or her work in the previous 2 weeks with respect to the self-selected behavior goal and action plan, and then created a new goal or modified existing goals. If a goal was not achieved, the coach helped the participant identify and make a plan for overcoming barriers to achieving the goal. During the 6 months of the study, if the coach was unable to contact a participant three times according to the scheduled coaching agenda created by the participant, the coach would send a phone message and/or email to ask the participant whether he or she wished to stop coaching or drop out of the study.

In general, during the coaching, the coach used powerful open-ended questions, active listening, and direct communication skills to enhance patients' behavior change motivation, and then assisted the patient in setting their own behavior change goal and action plan. During the tracking session, the same skills were used to assist the patient in overcoming obstacles to change in behavior or create new behavior goal.

#### Control Arm: Usual Care Only

Each participant in the control group would only receive a face-to-face coaching session at the baseline. No coaching calls or goal setting were carried out with control group participants.

Both the intervention and control groups received diabetes health education and usual care based on the diabetes shared care network program of Cathay General Hospital after baseline testing. The diabetes health education was executed by diabetes educators instructing patients to comply with medical instructions, such as self-monitoring of blood glucose, taking medications, participating in regular physical activity, maintaining an appropriate diet, taking foot and eye care, and etc. All participants were allowed to call diabetes educators during the study period to ensure adequate educational resources. In addition, since health coach was not responsible for providing any health education during the intervention, patients who had enquiries were referred to the educators.

#### Outcome Measures

The main outcome variables were HbA1c level and self-efficacy of diabetes self-management. HbA1c was measured using the participant's regularly scheduled blood test. Diabetes self-efficacy of diabetes self-management was assessed by a validated scale—the Perceived Diabetes Self-Management Scale (PDSMS) (29). The scale includes eight items: four positive situations such as “I succeed in the projects I undertake to manage my diabetes” and four negative situations such as “No matter how hard I try, managing my diabetes doesn't turn out the way I would like.” Every question is rated on a Likert five-point scale, ranging from “strongly agree” (5 points) to “strongly disagree” (1 point). The total points of PDSMS range from 8 to 40, and the higher points means the higher self-efficacy. The Chinese version of the PDSMS was translated by Wu and it demonstrated high consistency and reliability (Cronbach's α = 0.926), high intra-class correlation coefficient (0.966), and high content validity (all 8 items were higher than 0.75 according to the content validity index) (30).

The second outcome was assessed using the Godin leisure-time physical activity scale (31, 32). It marked the number of days in a week the participants did vigorous, medium and/or light physical activities, such as “During a typical 7-day period, how many times on the average do you do strenuous exercise for more than 15 min during your free time?” After weighing and summing up each level of physical activity, the higher the figure the more physical activities the participant engaged in. We did not design dietary test in this study since it is a complex behavior to detect its change within a small group.

The sociodemographic variables include gender, age, and educational level, and other variables are body mass index (BMI), systolic blood pressure (SBP), diastolic blood pressure (DBP), duration of diabetes treatment at Cathay General Hospital, insulin therapy use, and the health education participation in past 2 years.

### Statistical Analysis

Chi-square tests or *t*-tests were used to assess differences in sociodemographic factors, self-efficacy of diabetes self-management, physical activity behavior, and HbA1c between the two groups. Paired-t test was used to assess the difference in HbA1c, self-efficacy of diabetes self-management and physical activity for each group. In order to minimize the probable potential difference that might cause type I error which arose from the different groups the participants wished to join in, analysis of covariance (ANCOVA) was adopted to analyze the difference of HbA1c, self-efficacy of diabetes self-management and physical activity between the groups to account for the possible random effects. In addition, we also adopted the same method to compare the difference between pre-test and the index of these three outcomes. Finally, the repeated-measures ANOVA test was used to test for mean difference between the two groups on HbA1c, physical activity and self-efficacy of diabetes self-management outcomes at baseline, 3 and 6 months. We estimated the intraclass correlation coefficient between baseline HbA1c with other variables to detect whether potential variables were significant relating to HbA1c and had a significant difference between these two groups for ANCOVA adjustment. Since educational level and the duration of diabetes treatment at Cathay General Hospital were different between the two groups, we adjusted them for ANCOVA and repeated-measures ANOVA (Table 1). We also adjusted baseline value in these two analyses. Intention-to-treat analysis was not used since the ethical policy stated that non-compliers who refused to continue to participate had to be excluded from the analysis. All tests were analyzed at a 95% significance level (p < 0.05). The analyses were conducted using PASW 20.0 software for Windows (SPSS, Chicago, IL).

**Table 1:** Demographic characteristics and baseline value of study groups.

	**Demographic characteristics N (%)**		***p*-value**
	**Intervention group (*n* = 58)**	**Control group (*n* = 56)**	
Gender			0.403
Male	23 (39.7)	18 (32.1)	
Female	35 (60.3)	38 (67.9)	
Educational level			0.015^**^
Junior high school and bellow	9 (16.4)	23 (43.4)	
Senior high school	21 (38.2)	17 (32.1)	
University	22 (40.0)	12 (22.6)	
Master's degree or above	3 (5.5)	1 (1.9)	
Age, years (mean ± SD)	57.72 ± 11.18	60.54 ± 13.00	0.218
Insulin therapy			0.864
Yes	26 (44.8)	26 (46.4)	
No	32 (55.2)	30 (53.6)	
BMI (mean ± SD)	26.21 ± 5.39	27.24 ± 6.57	0.376
SBP (mmHg, mean ± SD)	134.39 ± 16.54	137.67 ± 22.31	0.390
DBP (mmHg, mean ± SD)	76.34 ± 17.84	72.59 ± 21.18	0.317
Health education participation in past 2 years (times,!!! mean ± SD)	0.81 ± 0.98	0.77 ± 0.69	0.790
Duration of diabetes treatment in CGH (years, mean ± SD)	7.36 ± 3.17	5.81 ± 3.90	0.022^*^
HbA1c (%,mean ± SD)	8.90 ± 1.43	8.51 ± 1.25	0.125
Physical activity points!!! (mean ± SD)	14.64 ± 16.00	15.00 ± 15.73	0.905
Self-efficacy of diabetes self-management points!!! (mean ± SD)	24.21 ± 4.12	25.02 ± 4.25	0.340

* *0.01 < p < 0.05*,

** *p < 0.01*.

*SD, standard deviation*.

*CGH, Cathay General Hospital*.

## Results

### Baseline Data

**Figure 1:**
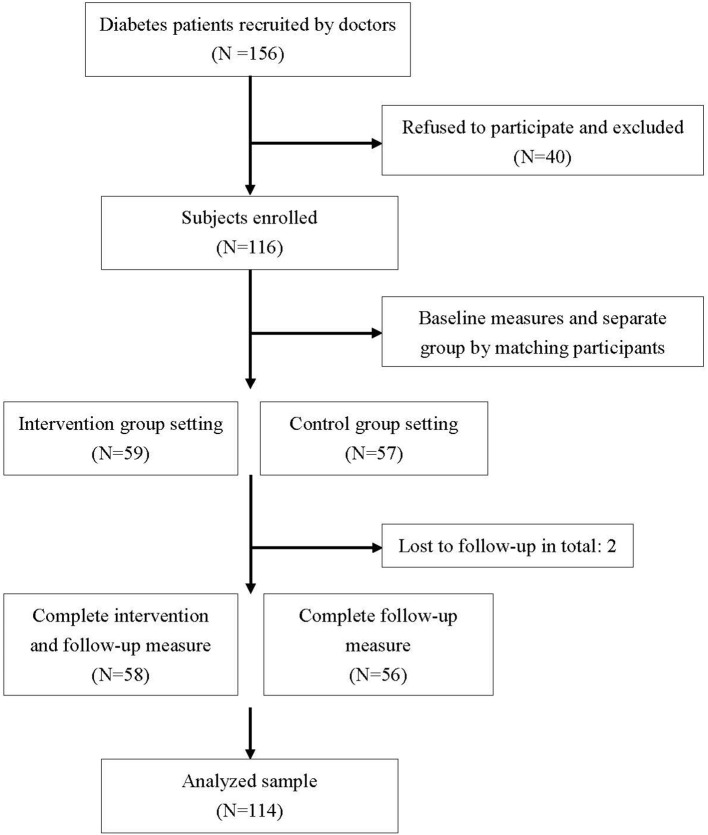
Flow diagram of participants: recruitment, intervention and follow-up.

Between June and October 2017, two physicians had invited 156 potential penitents to participate and eventually a total of 116 subjects enrolled in the study, resulting in a recruitment rate of 74%, and outcome measures were available for 58 patients (98% of the 59 patients) in the coaching intervention group and for 56 patients (98% of the 57 patients) in the control group (Figure 1). Two participants withdrew from the study due to personal reasons. Due to the non-participants who did not sign the informed consent, we were unable to collect data for comparing the difference in characteristics between patients who participated or not. Besides, due to the IRB's ethical policy, two withdrawers' data were excluded from the analysis. Most coaching contact was carried out by phone, but some sessions were completed with face-to-face meetings. In all, 58 participants in the intervention group completed 93.25 h of coaching, including the first session and the five follow-up sessions. On average, the participants in the control group spent less time in the first session than the participants in the intervention group, simply because some just left after they rejected the coach's help.

The demographic characteristics of the study groups are listed in Table 1. Of 114 participants, 64% were female, mean age was 59.1 years (*SD* = 12.1), only 33.8% had a bachelor's degree or higher, mean BMI was 26.73 (*SD* = 6.00), mean SBP was 136.00 mmHg (*SD* = 19.56), mean DBP was 74.50 mmHg (*SD* = 19.55), mean duration of diabetes treatment at Cathay General Hospital was 6.60 years (*SD* = 3.62), and the mean time of health education participation in past 2 years was 0.79 times (*SD* = 0.85). On average, the participants in the control group had a significant lower education level (*p* = 0.015) and a shorter duration of diabetes treatment at this hospital (*p* = 0.022), but gender, age, BMI, SBP, DBP, health education participation and insulin therapy were of no significant difference between the two groups at the beginning of the project. At baseline, the average HbA1c of the total 114 participants was 7.1% (*SD* = 1.35) and there was also no significant difference between these two groups (*p* = 0.125).

### Coaching Intervention Outcomes

**Figure 2:**
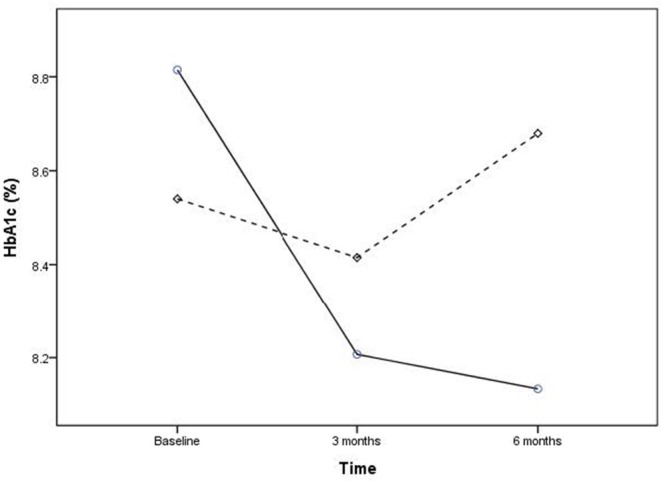
Effect of intervention within 6 months on HbA1c. Intervention group: solid line, control group: dashed line.

The range of changes in HbA1c during the study period were −3.0 to +1.0% in the intervention group and −3.2 to +1.7% in the control group. Overall, 67.2% of participants in the intervention group and 37.5% in the control group had a decrease in HbA1c levels within 6 months (not shown in the table). The coaching intervention was associated with a significant decrease of 0.64% (CI = 0.45 to 0.83) in HbA1c level within 3 months (*p* < 0.01) and a decrease of 0.68% (CI = 0.40 to 0.96) within 6 months (*p* < 0.01); a non-significant decrease of 0.09% (CI = −0.15 to 0.33) in HbA1c was observed in the control group within 3 months (*p* = 0.437) and an increase of 0.14% (CI = −0.42 to 0.15) within 6 months (*p* = 0.352) (Figure 2). The ANCOVA test revealed HbA1c was significantly different between these two groups at 3- and 6-months follow-up measure (*p* < 0.01) (Table 2). The repeated-measures ANOVA analysis revealed that the coaching intervention had significant effect on the decrease in HbA1c within 6 months (p < 0.001).

**Table 2:** Effectiveness of coaching intervention of study groups according to ANCOVA test and repeat-measured ANOVA test.

	**Effectiveness of coaching intervention (mean** **±** **SD)**		***p*-value for group difference**
	**Intervention group (*n* = 58)**	**Control group (*n* = 56)**	
HbA1c, %			
Baseline	8.90 ± 1.43	8.51 ± 1.25	0.125
3 months	8.26 ± 1.34^a^	8.42 ± 1.35	0.003^**^
6 months	8.20 ± 1.27^a^^,^^b^	8.61 ± 1.26	<0.001^**^
Physical activity points			
Baseline	14.64 ± 16.00	15.00 ± 15.73	0.905
3 months	20.16 ± 15.27^a^	14.63 ± 15.67	0.003^**^
6 months	18.79 ± 14.66^a^^,^^b^	10.52 ± 9.20	<0.001^**^
Self-efficacy of diabetes self-management			
Baseline	24.21 ± 4.12	25.02 ± 4.25	0.340
3 months	27.53 ± 4.51^a^	25.29 ± 4.45	0.006^**^
6 months	28.55 ± 4.89^a^^,^^b^	25.34 ± 4.24	0.008^**^

^*^0.01 < p < 0.05,

** *p < 0.01*. *HbA1c, hemoglobin A1c; SD, standard deviation*.

a *Significant difference in difference between groups*.

b *Significant difference in difference over 6-month intervention period between groups in the repeated measures ANOVA*.

There were 75.9% of participants in the intervention group and 46.4% in the control group had an increase in self-efficacy of diabetes self-management within 6 months. An obvious increase of 3.04 points (CI = −4.46 to −1.60) in self-efficacy of diabetes self-management was observed within 3 months (*p* < 0.01) and an increase of 3.89 points (CI = −5.60 to −2.17) within 6 months (*p* < 0.01) in the coaching group, whereas there was a non-significant increase in the control group within 3 and 6 months. The ANCOVA test revealed self-efficacy of diabetes self-management was significantly different between these two groups at 3- and 6-months follow-up measure (*p* < 0.01). The repeated-measures ANOVA analysis revealed that the coaching intervention had a significant effect on the increase in self-efficacy of diabetes self-management within 6 months (*p* = 0.020).

There was a significant increase in physical activity within both 3 (*p* < 0.01) and 6 months (*p* = 0.02) in the coaching group, but none in the control group. The ANCOVA test revealed physical activity was significantly different between these two groups at 3- and 6-months follow-up measure (*p* < 0.01). The repeated-measures ANOVA analysis revealed that the coaching intervention had a significant effect on the physical activity within 6 months (*p* = 0.001).

Moreover, both educational level and the duration of diabetes treatment at Cathay General Hospital were non-significant in all multi-variable analysis on HbA1c, physical activity and self-efficacy of diabetes self-management.

## Discussion

This is the first health coaching study trial conducted in a medical center in Taiwan. The study indicated that a 6-month health coaching by a professional coach reduced HbA1c in a group of type 2 diabetes patients by ~0.68%. In general, the control group had a lower level of education, comparing to the intervention group; however, the education level difference did not have a significant effect on HbA1c. The control group neither achieved a significant decrease in HbA1c nor gained an increase in physical activity or self-efficacy of diabetes self-management. Our findings supported those of most previous diabetes health coaching studies. For example, the review by Sherifali et al. reported that health coaching could reduce HbA1c by ~0.23 to 0.57% within 6 months (5). However, most diabetes health coaching research studies are of moderate to low quality (5). It is difficult to discern whether our coaching was better than the coaching in other studies since all of the studies included different frequencies, delivery methods, and qualities of coaching (5, 14, 17, 33–36).

Our second outcome were physical activity and self-efficacy of diabetes self-management which had a direct link to the benefit of health coaching. In the intervention group, the participants showed higher levels of physical activity, in terms of the number of times and/or the level of intensity they engaged in, and an increase in self-efficacy of diabetes self-management. Our results agreed with those of diabetes health coaching studies (17, 23, 26, 37). Physical activity is not the only factor that affects blood sugar level, other health behaviors, such as diet and self-monitoring on glycemic control, also play a part. We will include these behaviors in our future research. At present, few studies are done on how health coaching has the impact on the frequency of self-monitoring on glycemic control among the diabetics, and this should be included as one of the researches focuses in the future.

Despite the fact that quite a few studies have indicated how health coaches can benefit the self-management on chronic diseases and improve the health of patients as mentioned; however, there are several considerations to be noted regarding the promotion and application of this study. First, there is no “golden standard” in the frequency and length of coaching intervention, and this makes comparisons among coaching intervention studies difficult. Wolever et al. had published a review study to define the role of a “health and wellness coach.” It was noted that the average length of a health coaching session was 35.8 min, but the range was 5 min to 2.5 h depending on the needs of the client/patient (4), and that the frequency of coaching sessions fell in the weekly to monthly range. In real-world settings, coaching session frequency and length are decided by both the coach and the client as they enter a signed coaching agreement. In addition, unlike general motivational interview studies which could test research fidelity through tools like Motivational Interviewing Treatment Integrity (MITI) (38), health coaching is not fully applicable to MITI testing since the definition of coaching is more complicated. Therefore, we believe that international licenses and training with strict standards like ICF can enhance the fidelity of implementation and quality of such research, also, the feasibility of implementation in the real world. In addition to the time spent and the coach's competence, the modality of coaching (e.g., face-to-face, telephone, network, or mixed) is also varied among the literature. There is no research to compare or suggest which kind of coaching modality is better until now. From the literature review, more studies used face-to-face mixed with telephone coaching (6, 7, 39, 40). We believe that considering the health coaching is patient-centered, it is reasonable to have the coach communicate with patients and adopt the most appropriate approach and modality to better implement the intervention and reduce barriers to participate. Although this feature is not conducive to rigorous research evidence comparison, it can be as close as possible to reality and provide a reference for real-world applications.

In this study, it was estimated that the coach spent 93.25 h on 59 participants in 6 months, including the one who withdrew from the study. At present, since most studies adopt different cost calculation (e.g., telephone coaching charges, hourly labor costs, medical costs, etc.), therefore we were unable to directly compare with other studies to determine if this study has sufficient cost-effectiveness or positive returns on investment (ROI). Until now, most health coaching cost-effectiveness studies reported Health coaching has higher ROI than general care alone (41), and it might decrease the morbidity associated with diabetes-related complications, as well as reduce medical costs associated with diabetes and increase quality-adjusted life-years (42–44). However, these articles also noted that after the intervention ends, the benefit may decrease overtime, and it is suggested that health coaching programs require careful tracking of outcomes and additional as-needed coaching sessions every 3 to 6 months (20).

Apart from the main study, due to a degree of heterogeneity of health education to these patients (e.g., different topics, varied time spent, etc., depending on the needs of the patients), we did not adjust variables about health education in our outcome analysis but only briefly calculated and found the intervention group participants who utilized the health education resource had a better coaching effect in HbA1c by 0.3% within 6 months, though non-significant statistically (not shown in table). This might mean that health coaching did assist patients in finding their own problems more effectively and in realizing the need for health education in the process of their lifestyle change, and thereby enhancing the coaching effectiveness. The current practice of diabetes-shared care in our center is to provide more education resources for patients with poor glycemic control and poor self-efficacy of diabetes self-management. We have found that in the sample of this study (*n* = 114), the patients who did receive health education in the past 2 years had a worse glycemic control (HbA1c = 9.07%) than those who did not receive health education (HbA1c = 8.32%) at baseline. The health education was seemingly ineffective and inadequate to those participants if they were not involved in other health behavior change program. This might mean that for some patients with poor glycemic control, the current diabetes-shared care service in Taiwan is not appropriate enough. Before the education referral, the health coaching can be used to clarify the patient's problems and help the current shared care system to provide more effective educational resources.

Our study has several strengths. First, we used an ICF-credentialed coach, which promised high-quality coaching. It is rare within the field of health coaching studies to include a coach who holds a specific coach credential. Most health coaching studies have not explained their coaches' training qualities and/or skills tests, and some studies even included people who were new to coaching after only a few hours of training. The coach's ability is a potential limitation to these kinds of studies, but this factor tends to be ignored in those reports. Second, that only 2 participants withdrew within 6 months might reflect the patients' high level of loyalty to the hospital, or it might also be the representation of the quality and effectiveness of coaching that was acceptable to the patients. Since effective coaching can really help with medical communication with patients (45, 46), this should reduce patients' withdrawal rate.

The limitations of this study could be used to build future research. First, the quasi-experimental design reduced quality of evidence in this study on account of the potential selection bias. In this study, since we were unable to obtain a database of the population to compare whether our sample was significantly different from the population, we could only adopt statistical control methods and interpret the results of this study in a more conservative way. Education level and the duration of diabetes treatment at Cathay General Hospital were the only significant difference between intervention and control group at baseline, and both of them were non-significant in multiple analysis. Hence, we could believe that these two groups might not have significant selection-bias in this study. Based on this, we suggest that future research in health coaching in Taiwan should involve more hospitals and then randomize participants at hospital level or implement randomization after screening potential eligible patients before recruiting them. In fact, other studies in the past have chosen to use quasi-experimental design rather than randomized controlled trials in order to increase the enforceability of the study (47, 48). Second, we used only physical activity as a test for health behaviors in this study, but there are several behaviors that participants might select to change; this decreased our ability to analyze the effects of coaching on other behaviors. Therefore, we suggest that future research should include additional behavior tests with good reliability and validity. In addition, the goals of behavior change set by the participants might also be related to the effectiveness of the coaching program, but until now, there has been little literature on the impact of different goals on outcomes, so this may be one of the future research issues.

In addition to conducting more rigorous randomized controlled trials, for the purpose of implementing health coach research and services in the future, we suggest that there should be more research using certified coaches for training and a clear description of coaching ability assessment of interventionists. Also, more studies on the time and length of health coaching intervention are also needed. Second, the quality of coach training and background of the coach should be well-defined for more viable health coaching service. Until now, most health coaching studies have quite large different training hours ranging from 2 h to 2 years with median 40 h; also, most of them do not mention their certification and training evaluation standards, not even mentioning the source or quality of trainers (4). Whether it is for the medical staff to receive coach training or professional coaches to receive training related to health care, when applying the coach in the research, the training quality, competency standards and assessment methods should be clearly adopted and described in order to ensure the quality of coach intervention.

For the future applications in Taiwan, we suggest more health coaching studies be conducted to improve medical quality and chronic diseases prevention and management. As Taiwan's current chronic disease management is still mainly based on hospitals above the regional hospital level, it is not suitable for implementation in primary care institutions, so it might still be a regional hospital or a medical center as the main testing site in the short term. Though coaching intervention has a heterogeneous characteristic, it is worthwhile to combine with the diabetes-shared care network in Taiwan. In addition, it can also manifest the efforts of the shared decision making (SDM) that has been promoted in recent years. In general, health coaching services can be integrated into outpatient clinics or introduced as a part of health education program. Hence, more health coaching studies are needed to reinforce the applicability of the diabetes health coaching and the development of other health coaching disciplines in Taiwan.

## Conclusions

On top of current “Diabetes Shared Care Network” and standard diabetes treatment in Taiwan, we found that 6 months of health coaching may be an effective strategy to improve HbA1c and self-efficacy. Based on the limitations and strengths of this study, we suggest that more studies with designs that yield higher quality evidence for the role of health coaching in diabetic patients are needed in Taiwan.

## Data Availability Statement

The datasets during the current study are not publicly available in order to ensure the protection of the informants anonymity but are available from the corresponding author on reasonable request and must comply with Institutional Review Board of Cathay General Hospital's rule.

## Ethics Statement

This study was approved by the Institutional Review Board of Cathay General Hospital (Ref no. CGH-OP106001). A written informed consent was taken from each respondent before conducting the study.

## Author Contributions

C-LL, R-YC, C-TS, and Y-TC participated in the conception and design of the study. Y-TC, L-CH, C-LChu, and C-LCha participated in acquisition of data. R-YC, C-LL, L-CH, and Y-TC performed the statistical analysis and drafted the manuscript. All authors read and approved the final manuscript.

### Conflict of Interest

The authors declare that the research was conducted in the absence of any commercial or financial relationships that could be construed as a potential conflict of interest.

## Abbreviations

WHO: World Health Organization
ICF: International Coaching Federation
ACC: Associate Certified Coach
HbA1c: Hemoglobin A1c
BMI: Body mass index
SBP: Systolic blood pressure
DBP: Diastolic blood pressure
IRB: Institutional Review Board
PDSMS: Perceived Diabetes Self-Management Scale
ANOVA: Analysis of variance
ANCOVA: Analysis of covariance
SD: Standard deviation
MITI: Motivational Interviewing Treatment Integrity
SDM: Shared decision making.
